# Divergence in Coding Sequences and Expression Patterns Among the Functional Categories of Secretory Genes Between Two Aphid Species

**DOI:** 10.3390/biology14080964

**Published:** 2025-08-01

**Authors:** Atsbha Gebreslasie Gebrekidan, Yong Zhang, Julian Chen

**Affiliations:** 1 State Key Laboratory for Biology of Plant Diseases and Insect Pests, Institute of Plant Protection Chinese Academy of Agricultural Sciences, Beijing 100193, China; atsbha1415@gmail.com; 2 Tigray Agricultural Research Institute, Mekelle 7000, Ethiopia

**Keywords:** rose grain aphid, coding sequence, gene expression, pleiotropy, secretory gene

## Abstract

Disparities in the functional classification of secretory genes among aphid taxa may be attributed to variations in coding sequences and gene expression profiles. However, the driving factors that regulate sequence evolution remain unclear. This study aimed to investigate the differences in coding sequences and expression patterns of secretory genes between the rose grain aphid (*Metopolophium dirhodum*) and the pea aphid (*Acrythosiphon pisum*), with a particular focus on their roles in evolutionary adaptations and functional diversity. The study involved the rearing of aphids, RNA extraction, de novo transcriptome assembly, functional annotation, secretory protein prediction, and comparative analysis of coding sequences and expression patterns across various functional categories using bioinformatics tools. The results revealed that metabolic genes exhibited greater coding sequence divergence, indicating the influence of positive selection. Moreover, significant expression divergence was noted among functional categories, particularly in metabolic and genetic information processing genes, which exhibited higher variability. This study enhances our understanding of the molecular mechanisms that contribute to phenotypic and genetic diversity among aphid species. This study elucidates the relationship between variations in coding sequences and differences in gene expression among functional categories, thereby establishing a foundation for future studies on gene evolution in response to environmental pressures.

## Simple Summary

The genetic factors influencing the variation in host preference among aphid species remain poorly understood. In this study, we focused on the secretory genes of the rose grain aphid and the pea aphid to explore the molecular basis of the sequence and expression variation observed between the two aphid species. Our analysis involved examining the coding sequences and expression patterns of the secretory genes in these species. The results indicate that differences in both the coding sequences and expression levels of secretory genes related to metabolism are essential in shaping host preference and adaptation disparities between these aphid species.

## 1. Introduction

Aphids are small insects with soft bodies that inhabit various environments worldwide. They belong to the superfamily Aphidoidea, which is believed to have evolved approximately 280 million years ago and is classified under the order Hemiptera. Aphids feed on plant sap using their piercing-sucking mouthparts, which can cause damage to plants by stunting growth, curling leaves, and transmitting plant viruses when sucking the phloem sap from the plant [1,2,3]. There are substantial differences among aphid species in host preferences with the presence of alternative food sources. Such differences in host preferences could be a result of numerous factors, but differences in host preference likely play the most important role in abundance, reproduction, and dominance in biological and ecological niches. However, the regulatory mechanisms conserved among aphid species are not clearly understood. Unlike *Acrythosiphon pisum*, *Metopolophium dirhodum* belongs to the larger tribe of Macrosiphini in the family Aphididae. 

Divergence in coding sequences and expression patterns plays a crucial role in the functional evolution of genes, especially those involved in species-specific adaptations [4]. In the context of aphids, understanding how these divergences manifest in secretory genes, particularly those related to immune functions, can provide insights into their adaptation to different host plants and environments [5]. Secretory genes, which encode proteins destined for secretion outside the cell, play an important role in various biological processes, including immune response, digestion, and intercellular communication [6]. Divergence in the coding sequences of these genes can lead to variations in protein structure and function, whereas differences in gene expression patterns can influence the timing and location of protein production [7]. Secretory proteins with positive selection have been identified as strong candidates for specific infestation strategies of aphid species on a few or many crops [8,9]. The examination of these two facets of genetic variation helps to gain insights into the molecular mechanisms driving phenotypic diversity and the evolutionary pressures that shape the functional landscape of secretory proteins. The study of gene expression and coding sequences in aphids’ aids in understanding their functional diversity and evolutionary adaptation. Aphids, as sap-sucking insects, have evolved complex interactions with their host plants, largely mediated by secretory proteins [10]. The differences in coding sequences and expression divergence of secretory genes between rose-grain and pea aphids might pave the way for studies of the molecular mechanisms that form the biological variation in functional categories between rose grain aphid and pea aphid species.

Genes encoding functional categories may differ in sequence or expression divergence because they differ from other factors corresponding to sequence or expression divergence [11]. Once these disparities are controlled, there are no differences in the sequence between the gene functional categories. Sequence divergence is generally negatively correlated with pleiotropy [12]. In addition to pleiotropy, differences in coding sequences are related to several other factors, particularly at the level of gene expression. Similarly, expression divergence is associated with pleiotropy and expression levels. Therefore, these factors could potentially make it difficult to understand any differences in sequences or expression differences between gene functional categories. However, to the best of our knowledge, the relative importance of gene functions and other factors for the coding sequence and the differences in the expression of putative effectors have not been explicitly addressed in most organisms.

This study investigated the distinctions between coding sequence and expression divergence in secretory genes linked to basic functional categories in the rose grain aphid (*M. dirhodum*) and pea aphid (*A. pisum*). This study aimed to determine how these variations affect the evolutionary trajectories of aphid species and their adaptability to environmental changes. This study used the rose grain aphid as a model insect to investigate variations in secretory genes in relation to non-secretory genes, meticulously organized according to KEGG pathways. Furthermore, it assesses the independence of differences across functional categories from other factors such as pleiotropy and expression levels. We also evaluated the evolutionary rates of genes and the selective pressures influencing the coding sequence evolution of secretory genes across functional categories. This was achieved using de novo transcriptome assemblies and expression data.

## 2. Materials and Methods

### 2.1. Rearing of the Rose Grain Aphid

The research commenced with the cultivation of apterous adult rose grain aphids (*M. dirhodum*) originating from a single, clonal lineage. These aphids were sustained on susceptible varieties of wheat (*Triticum aestivum* L. cv. Zhongmai-175), a member of the Poaceae family (grass family), which were grown in cages within a controlled environment. The rearing conditions were meticulously monitored to ensure optimal growth and reproduction of the aphid population. Specifically, the temperature was maintained at 18 °C, with a photoperiod consisting of 16 h of light and 8 h of darkness. Additionally, both seedlings and aphid populations were maintained at a relative humidity of 75–80%. Typically, two to three generations of the rose grain aphid are required to conduct a thorough examination and dissection of their heads and the associated salivary glands.

### 2.2. Tissue Dissection and RNA Extraction

Aphid cranial tissues, along with the associated salivary glands, were systematically excised from a cohort of 200 individuals under microscopic scrutiny while immersed in a 1% phosphate-buffered saline (PBS) solution. The freshly excised specimens were promptly preserved and subjected to freezing in liquid nitrogen to avert any potential degradation. Total RNA was extracted using TRIzol reagent [13], followed by purification using the Tianmobio TR150 Mini Total RNA Extraction Kit, in strict accordance with the manufacturer’s protocols. The concentration and quality of total RNA were evaluated using a nano spectrophotometer (IMPLEN, Munich, Germany). RNA integrity was confirmed by electrophoretic separation on a 1% agarose gel and further assessed using the RNA Nano 6000 Assay Kit on an Agilent Bioanalyzer 2100 system (Agilent Technologies, Santa Clara, CA 95051, USA). For subsequent applications, three micrograms of total RNA with a 260/280 optical density (OD) ratio between 1.8 and 2.1 and an RNA integrity number (RIN) of 7.5 were chosen for library preparation and Illumina sequencing. Paired-end sequencing was performed by Novo (Beijing, China) on the HiSeq2500 platform, utilizing HiSeq Control Software 2.2.58/RTA 1.18.64 with a sequencing setup of 2 × 126 and the HiSeq SBS Kit v4 chemistry.

### 2.3. Transcriptome Assembly and Prediction of Orthologous Genes

Following the sequencing process, the raw reads were meticulously trimmed using Trimmomatic with standard configurations [14]. The quality of the trimmed reads was assessed using FastQC version 0.11.5. De novo assembly of the trimmed reads was performed using Trinity [15], adhering to the default parameters. CD-HIT version 4.6.8 [16] and TransDecoder [17] were employed to cluster contigs exhibiting a minimum of 95% similarity and to predict coding sequences, respectively. To rectify potential assembly errors, the reads were remapped to the pre-filtered contig, and transcripts with Transcripts Per Million (TPM) values falling below 1 were excluded from further analysis. RNA sequencing yielded approximately 47.5 million raw reads, of which approximately 46.2 million were classified as clean. Ultimately, 31,344 transcripts and 18,030 unigenes were assembled from the transcriptomic sequences of the head tissue and salivary glands. To elucidate the orthologous secretory genes between the rose grain aphid and the pea aphid, a reciprocal best hit BLASTp analysis was performed using the predicted amino acid sequences derived from the rose grain aphid. Hits with an e-value of less than 1 × 10^−5^ were selected to develop a putative one-to-one ortholog matrix with respect to the pea aphid. The annotation of these orthologous sequences was facilitated using BLASTx (version 2.6.0), which aligned transcripts from the rose grain aphid with the protein-coding genes from the pea aphid.

A total of 5850 orthologous genes were retrieved, receiving consistent annotations against the non-redundant protein databases of pea aphids. To identify candidate secretory proteins, sequences containing signal peptides were extracted from the translated peptide sequences of the transcriptome using SignalP-6. Transmembrane domains within proteins possessing signal peptides were identified using the Transmembrane Hidden Markov Model (TMHMM). Proteins bearing signal peptides and either zero or one transmembrane domain were designated as candidate secretory proteins. In total, 705 secretory genes were predicted to encode secretory proteins from the assembled gene pool (18,030). Among these, 100 gene transcripts were identified as structurally and functionally conserved (orthologous) between the rose grain and pea aphids (Table 1).

### 2.4. Gene Expression Quantification

Transcriptomic sequences from the rose grain aphid were aligned to their filtered de novo assemblies using RSEM [18]. We extracted the read counts for the annotated orthologous secretory genes to form a comprehensive matrix, normalizing the data with the fragments per kilobase of transcript per million mapped reads (FPKM) method in EdgeR [19]. The FPKM values were then transformed using the Box–Cox method and assessed using the Jarque–Bera test to achieve normalization of the dataset [20]. An empirical distribution of the Box–Cox transformed FPKM values was generated using kernel density estimation in R version 4.3.2 [21]. Finally, Z-transformed FPKM values were calculated following the established protocols by Asar et al. [22], Dag et al. [23].

### 2.5. Interspecific Divergence of the Coding Sequences

To analyze coding sequence divergence, we aligned orthologous secretory gene sequences from the de novo transcriptome of the rose grain aphid with the longest transcript from the pea aphid using the ClustalW codon aligner algorithm within MEGA 11, employing the Kumar method with Kimura 2-para correction [24,25]. The coding sequences from the rose grain aphid transcriptome were supplemented with sequences from the pea aphid reference genome to improve the overlap in the datasets. Only genes with at least 150 base pairs of overlapping coding nucleotide sequences were included in the analysis. Divergence was quantified by calculating the number of nonsynonymous substitutions per nonsynonymous site (dN). We also calculated the number of synonymous substitutions per synonymous site (dS) to evaluate whether any differences in dN were due to variations in mutation rates or selective pressures [26]. The dNdS ratio was computed to assess the level of selective pressure on the coding sequences, with a higher ratio indicating either relaxed purifying or positive selection. For genes where both dN and dS equaled 0, a dNdS ratio of 0 was assigned. This indicates that these genes did not show any variation in coding sequences and, thus, did not undergo any selective pressure.

### 2.6. Gene Expression Variation Among Functional Categories

The standard deviation of the zFPKM values was used as a measurable parameter to assess the intraspecific variability in gene expression. The methodology established by Chen et al. [27] was rigorously followed to ascertain the divergence in gene expression, employing the smatr package within the R programming environment to calculate the residuals originating from the standardized major axis regression. This regression analysis measured the mean expression levels of orthologous secretory genes in relation to the average expression levels of non-orthologous secretory genes across the functional categories.

### 2.7. Functional Protein Association Network (Pleiotropy)

Pleiotropy was considered a covariate in the assessment of variations in the coding sequence and expression divergence among functional gene categories. The number of protein–protein interactions (PPI) served as a proxy for estimating pleiotropy. The PPI counts for *M. dirhodum* orthologs of *A. pisum* genes were retrieved from a non-redundant protein database (https://string-db.org/ retrieved on 25 March 2024), adhering to a confidence threshold of ≥0.4.

### 2.8. Data Manipulation and Analysis

**Figure 1:**
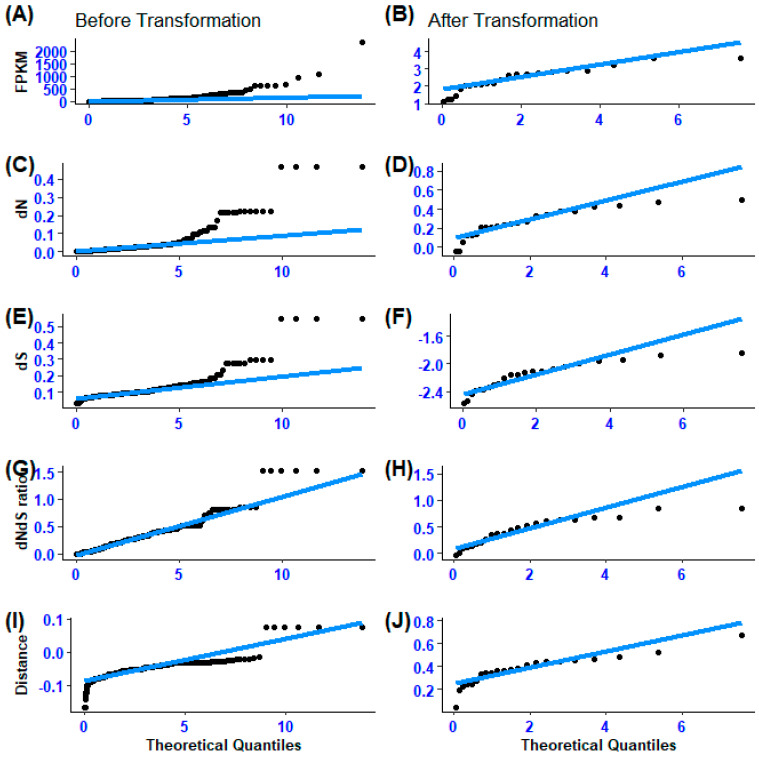
(**A**–**J**) Illustration of gene expression divergence (FPKM), non-synonymous substitution (dN), synonymous substitution, non-synonymous to synonymous substitution ratio (dNdS ratio), and the difference between non-synonymous and synonymous ratios (Distance) point data distribution before and after transformation.

Utilizing general linear models (GLM), a comparative analysis of orthologous secretory genes across five functional categories was performed, supplemented by Tukey’s and Dunnett’s post hoc tests. The GLM analysis was carried out using R-software (version 4.3.2), with expression data normalized by FPKM and subjected to Box–Cox transformation before analysis. To ensure a robust statistical evaluation of the effects of functional categories on gene expression, the continuous independent variables—dN, dS, dNdS ratio, and distance—were standardized to achieve a normal distribution (Figure 1). The input data used for calculating FPKM, dN, dS, dNdS ratio, and Distance were deposited in the NCBI SRA repository (http://www.ncbi.nlm.nih.gov/bioproject/PRJNA1134911 deposited on 12 July 2024) and on the Zenodo website (https://zenodo.org/uploads/13530906 deposited on 29 August 2024).

## 3. Result

### 3.1. Variation in Coding Sequences Between Secretory and Non-Secretory Genes

**Figure 2:**
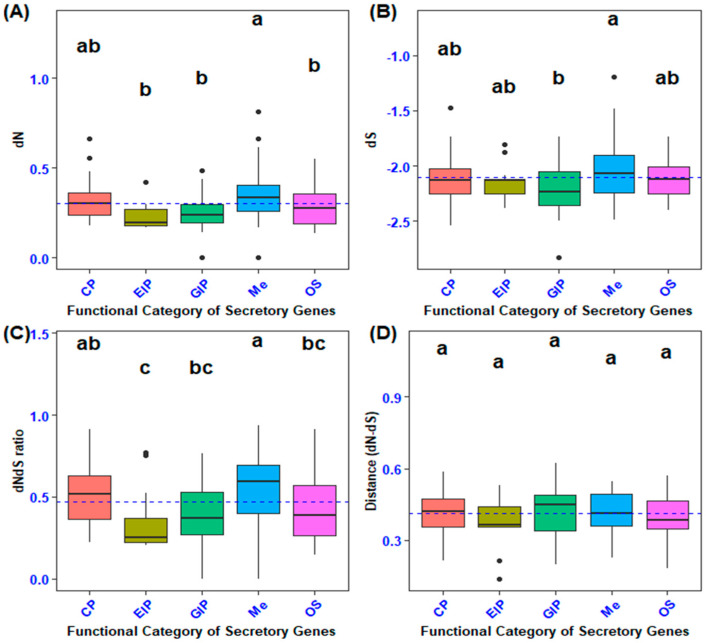
Comparison of coding sequence divergence in secretory genes across functional categories. (**A**) Pairwise analysis of the rates of nonsynonymous substitutions (dN) among various functional categories of secretory genes. (**B**) Pairwise analysis of the rates of synonymous substitutions (dS) among different functional categories of secretory genes. (**C**) Pairwise comparison of the ratio of nonsynonymous to synonymous substitutions (dNdS ratio) across functional categories of secretory genes. (**D**) Pairwise evaluation of the difference between the rates of nonsynonymous and synonymous substitutions (dN−dS) among the functional categories of secretory genes. Different lowercase letters indicate statistically significant differences among functional categories of genes, as determined by Tukey’s post hoc test.

**Figure 3:**
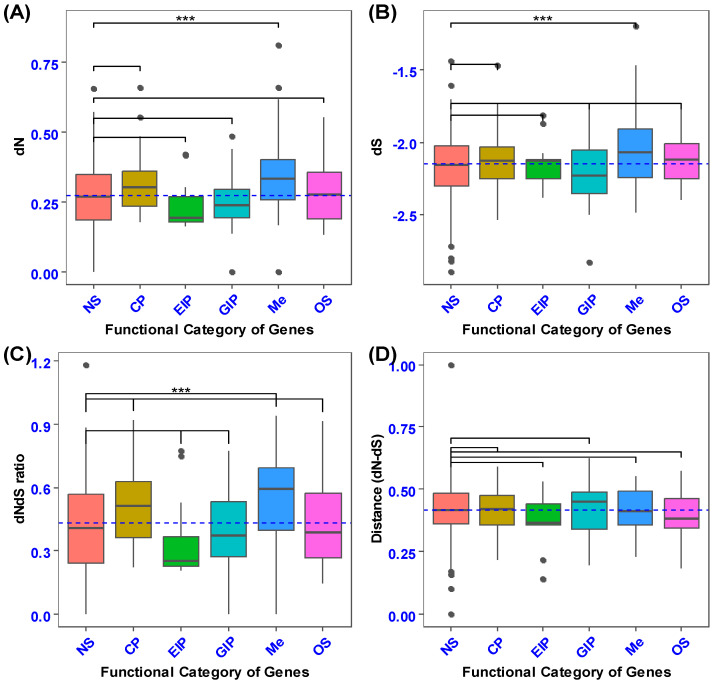
The box plot illustrates the comparison of coding sequence divergence between secretory and non-secretory genes across different functional categories. Panel (**A**) presents a pairwise comparison of the rates of nonsynonymous substitutions (dN). Panel (**B**) displays a pairwise comparison of the rate of synonymous substitutions (dS) between the secretory and non-secretory genes. Panel (**C**) shows the pairwise comparison of the rate of nonsynonymous substitutions relative to the rate of synonymous substitutions (dNdS ratio) between the secretory and non-secretory genes. Panel (**D**) highlights the pairwise comparison of the difference between the rate of nonsynonymous substitutions and the rate of synonymous substitutions (dN−dS) for these gene categories. Asterisks above the box plot indicate the functional categories of secretory genes that show statistically significant differences from the control (non-secretory) genes, as determined by Dunnett’s post hoc test: *** *p* < 0.001.

The mean coding sequence divergence (estimated as the rate of nonsynonymous substitutions; dN) differed among the functional categories of secretory genes (F4, 100 = 8.699, Tukey’s: *p* < 0.0001; Figure 2A). In particular, genes encoding metabolism (Me) had higher coding sequence divergence than those encoding cellular processes (CP), environmental information processing (EIP), genetic information processing (GIP), and organismal systems (OS) (Tukey’s: *p* < 0.0001). A comparison of the coding sequence divergence of secretory genes with 101 randomly selected non-secretory orthologous genes revealed that secretory genes involved in metabolism had higher nonsynonymous substitution rates (F5, 201 = 9.425, *p* = 0.0000000134, Dunnet’s: *p* < 0.0001; Figure 3A). The *p*-value of the secretory genes involved in metabolism was designated as 0.0001, and it was contrasted with the non-secretory genes that encoded the functional categories, which were the control group.

The examination of the synonymous substitution rate (dS, which serves as a measure of mutation rate) indicated that metabolic processes exhibited a higher dS than cellular processes, environmental information processing, genetic information processing, and organismal systems (F4, 100 = 3.733, Tukey’s: *p* < 0.01; Figure 2B). Variance analysis of the average synonymous substitutions in orthologs of secretory versus non-secretory genes demonstrated a highly statistically significant difference between the genes implicated in energy production and processing (metabolism) and the non-secretory ortholog genes (NS) across various functional categories (F5, 201= 5.34, *p* = 0.0000859, Dunnett’s: *p* < 0.0001; Figure 3B).

In the present investigation, the non-synonymous to synonymous substitution ratio (dNdS ratio) was quantified on gene sequences extracted from two phylogenetically proximate aphid species (*M. dirhodum* and *A. pisum*). This ratio serves as an indicator of structural configuration, intensity of recurrent selection, and pace of evolutionary change. The dNdS ratio metric exhibited relative insensitivity to the degree of natural selection. A comparative analysis of the non-synonymous to synonymous substitution ratios across various functional categories of orthologous secretory genes revealed that metabolism (Me) demonstrated a superior level of non-synonymous to synonymous substitution ratios (dNdS ratio) in contrast to genetic information processing (GIP), environmental information processing (EIP), and organismal systems (OS) (F4, 100 = 8.55, Tukey’s: *p* < 0.0001; Figure 2C). Moreover, cellular processes (CP) exhibited an elevated non-synonymous to synonymous ratio when juxtaposed with environmental information processing (*p* < 0.0001). In addition, secretory genes associated with metabolism exhibited an increased nonsynonymous-to-synonymous ratio relative to non-secretory orthologous genes (F5, 201= 7.665, *p* = 0.00000059, Dunnett’s: *p* < 0.0001; Figure 3C). Nevertheless, no statistically significant differences were observed between the mean values of non-synonymous and synonymous substitution ratios (dNdS ratio) across the functional categories of both secretory and non-secretory orthologous genes (Figure 2D and Figure 3D).

### 3.2. Mean and Variability of Gene Expression Divergence

**Figure 4:**
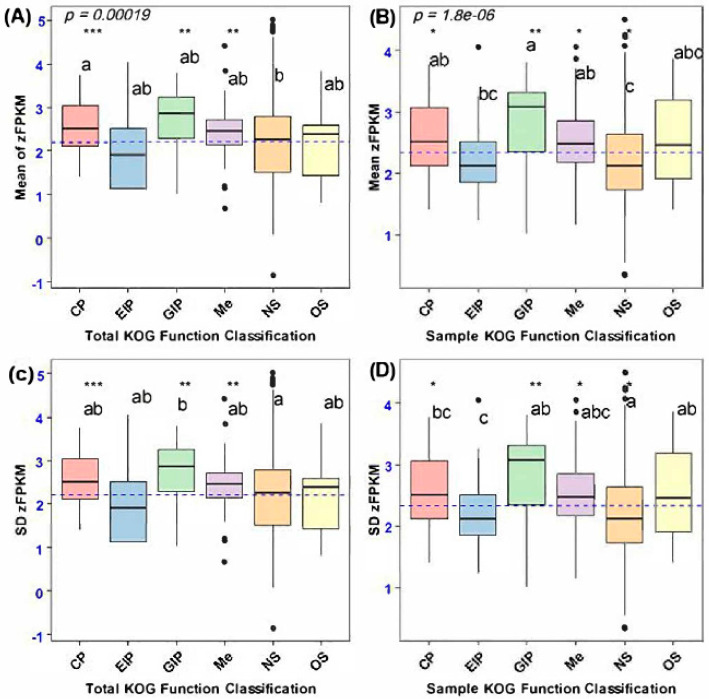
Mean and variation in gene expression (zFPKM) of secretory genes and a set of randomly selected non-secretory orthologous genes between functional categories. (**A**) Mean expression of total orthologous genes between functional categories. (**B**) Mean expression of non-secretory and secretory orthologous genes among functional categories. (**C**) Variance of zFPKM of total orthologous genes encoding different functional categories. (**D**) SD of zFPKM of non-secretory and secretory sample orthologous genes. Letters indicate which categories of immune genes were significantly different from each other based on Tukey’s post hoc test. Asterisks indicate the functional categories of secretory genes that were significantly different from those of non-secretory orthologous genes (NS) based on Dunnett’s post hoc test: *** *p* < 0.001, ** *p* < 0.05, * *p* < 0.05.

The frequency distribution of Box–Cox-transformed FPKM (zFPKM) values pertaining to the expression of both secretory and non-secretory (considered as control variables) orthologous genes demonstrated homogeneity, albeit with minor deviations observed in the mean and standard deviation of certain Box–Cox-transformed FPKM data points. Consequently, we implemented the Box–Cox standardization technique to recalibrate the secretory gene expression values to conform to a standard normal distribution before conducting the mean and variance estimation analysis [20,28]. An examination of the frequency distribution plot of zFPKM expression values indicated the absence of significant skewness associated with lowly expressed genes. Thus, we opted to retain 100 orthologous secretory and 101 non-secretory genes for coding sequence divergence analysis, rather than excluding genes characterized by low-expression values. Nonetheless, a statistically significant disparity in the mean zFPKM was observed across the functional categories of the orthologous genes (Figure 4).

The average expression levels exhibited variability among the orthologous categories of secretory genes, with genetic information processing demonstrating a significantly elevated expression level compared to environmental information processing (F4, 100 = 26.4, *p* < 0.0001, Tukey’s: *p* < 0.0001). Furthermore, secretory orthologous genes associated with genetic information processing (GIP), cellular processes, metabolism (Me), and organismal systems also exhibited a greater mean gene expression divergence relative to non-secretory orthologous genes (NS) (F5, 201 = 33.3, *p* = 0.0000018, Dunnett’s: *p* < 0.0001; Figure 4B). Notably, despite the observed variations in secretory genes pertaining to cellular processes, metabolism, and organismal systems, the expression divergence of secretory genes associated with genetic information processing was particularly accentuated compared to the expression levels of non-secretory orthologous genes. Nevertheless, the mean expression divergence of all orthologous genes varied among functional categories, wherein genes encoding cellular processes and genetic information processing exhibited greater gene expression divergence than non-secretory orthologous genes (NS) (F5, 2622 = 39, *p* = 0.0170, Dunnett’s: *p* < 0.0001; Figure 4A).

Intraspecific variation in gene expression, assessed through the standard deviation of zFPKM, revealed differences among the functional categories of total orthologous genes, particularly between secretory and non-secretory genes. Notably, non-secretory orthologous genes exhibited greater expression variability than those involved in genetic information processing (GIP), with statistically significant results (F5, 2622 = 37.6, *p* = 0.000092, Dunnett’s: *p* < 0.0001; Figure 4C). Furthermore, a significant difference in gene expression variance was found between randomly selected functional categories of non-secretory and secretory orthologous genes. Specifically, non-secretory orthologous genes showed higher expression variation than secretory genes associated with cellular processes and environmental information processing (F5, 201 = 6.683, *p* = 0.00000485, Dunnett’s: *p* < 0.0001; Figure 4D).

### 3.3. Coding Sequence and Expression Variation in Relation to Pleiotropy and Functional Gene Categories

**Figure 5:**
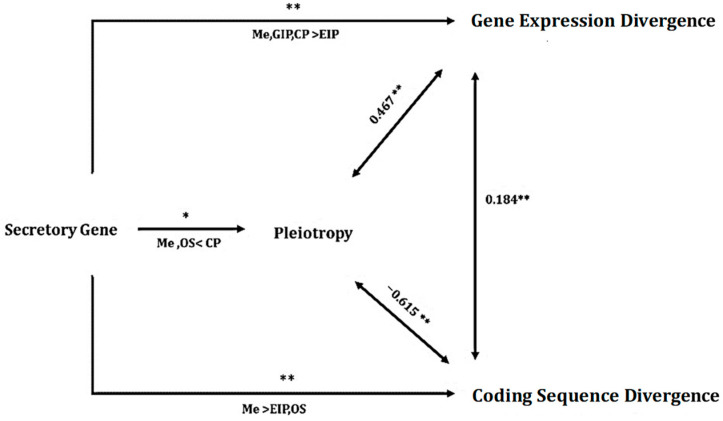
The path model illustrates the impact of the secretory gene category on three continuous variables: pleiotropy, divergence of gene expression, and divergence of coding sequence. It also shows the correlations between the variables. Asterisks above the arrows from the secretory gene category indicate the results of GLMs, whereas the text below the arrows indicates the results of Tukey’s post hoc tests. The abbreviations GIP, EIP, Me, CP, and OS represent genetic information processing, environmental information processing, Metabolism, Cellular process, and organismal system, respectively. The values at the bidirectional arrows represent Spearman’s rank correlation coefficients. ** *p* < 0.01; * *p* < 0.05.

A path model analysis was conducted to elucidate the relationships between coding sequence and expression divergence and various other factors, particularly focusing on the functional categories of secretory genes (SG), coding sequence divergence, gene expression divergence, and pleiotropy. In addition to the previously noted impacts on coding sequence and expression divergence, the functional category of secretory genes significantly influenced pleiotropy, as indicated by statistical analysis (GLM: F4, 100 = 4.54, *p* < 0.001; see Figure 5).

A generalized linear model (GLM) analysis was conducted to explore whether the differences in pleiotropy among various functional categories of secretory genes occurred independently of other factors. In this analysis, pleiotropy was designated as the response variable, whereas the functional category of genes served as the independent grouping variable for each outcome variable score (pleiotropy). In addition, gene expression divergence and sequence divergence were included in the model as continuous predictors. GLM analysis assessed pleiotropy in relation to the functional categories of secretory genes, expression divergence, sequence divergence, and three-way interactions involving gene functional categories, gene expression, and coding sequence divergence. The results revealed a negative association with coding sequence divergence (F1, 100 = 8.2, *p* = 0.03) and a positive correlation with gene expression divergence (F1, 100 = 5.2, *p* = 0.01). Notably, the difference in pleiotropy between genes associated with metabolic and organismal systems and those linked to cellular processes was significant (F4, 100 = 3, *p* < 0.05; Figure 5) when evaluated against genes related to environmental information processing and genetic information processing functional categories.

**Figure 6:**
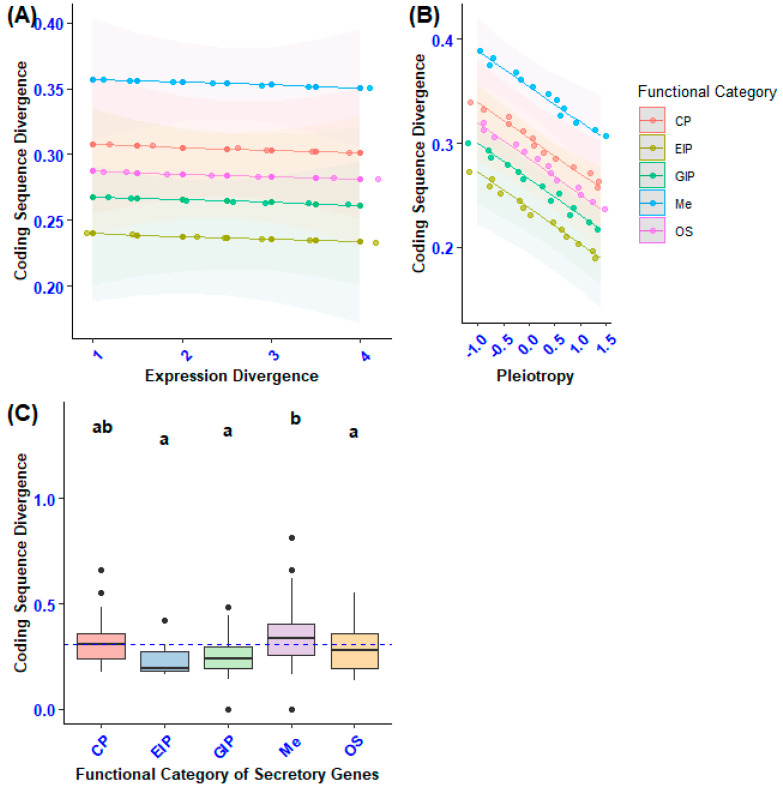
The diagram offers the following insights: (**A**) It demonstrates how expression divergence and the functional categories of secretory genes impact coding sequence divergence. (**B**) It underscores the effects of pleiotropy and the functional categories of secretory genes on coding sequence divergence. (**C**) It presents a pairwise comparison of coding sequence divergence (dN) among the functional categories of secretory genes, taking into account confounding variables. Statistically significant differences among gene functional categories are indicated by different lowercase letters, as determined by Tukey’s post hoc test.

To assess whether the variation in sequence divergence across the functional categories of secretory genes was influenced by the differences in gene categories, we conducted a Generalized Linear Model (GLM) analysis. This model was used to evaluate the effects of the functional categories of secretory genes, expression divergence, and pleiotropy on the divergence of gene sequences. Furthermore, we explored the potential impact of three-way interactions among gene category, expression divergence, and pleiotropy on coding sequence divergence. The results of our statistical analysis indicated a significant effect of the functional category of secretory genes on coding sequence divergence. Specifically, there was a notable difference in coding sequence divergence among secretory genes related to metabolism compared with those involved in environmental information processing, genetic information processing and organismal systems (GLM: F4, 100 = 6.797, *p* < 0.0001; Figure 6C). This finding remained significant even when accounting for the negative association between sequence divergence and pleiotropy (F1, 100 = 13.3, *p* = 0.01) as well as the positive interaction between coding sequence divergence and gene expression divergence (F1, 100 = 4.84, *p* = 0.0062).

**Figure 7:**
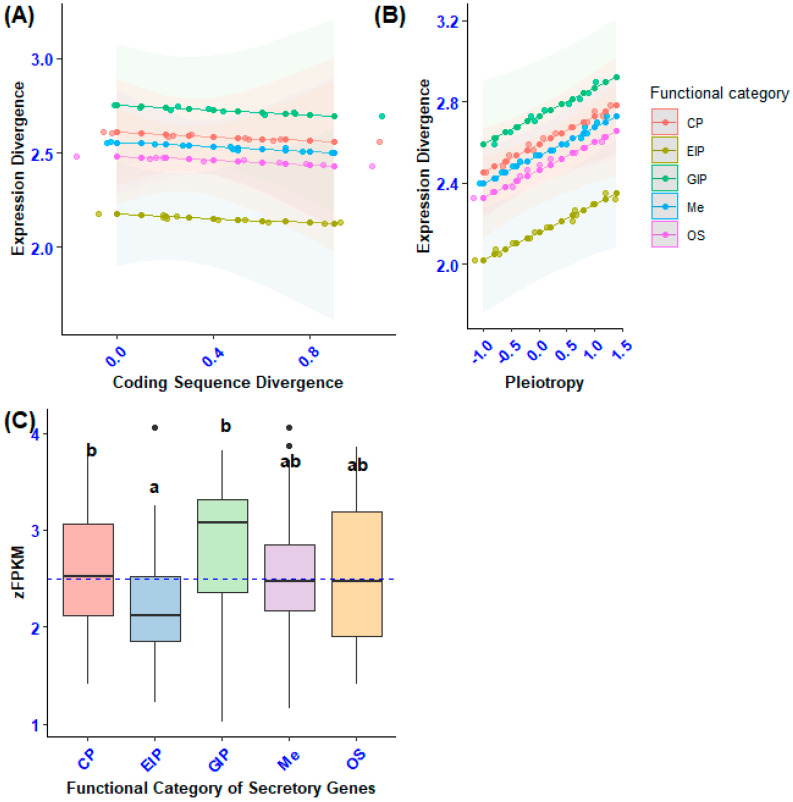
The diagram presents the following information: (**A**) The impact of coding sequence divergence and functional categories of secretory genes on the expression divergence of secretory genes. (**B**) Effect of pleiotropy and functional categories of secretory genes on the expression divergence of secretory genes. (**C**) A pairwise comparison of expression divergence (zFPKM) across functional categories of secretory genes, considering confounding factors. Statistically significant differences among gene functional categories are indicated by different lowercase letters, as identified by Tukey’s post hoc test.

To determine whether the variation in gene expression divergence among different categories of secretory genes was influenced by other factors, we conducted a Generalized Linear Model (GLM) analysis. In this analysis, we designated expression divergence as the dependent variable, with the gene category serving as the independent variable. We also included pleiotropy and sequence divergence as continuous predictors. Furthermore, we assessed three-way interactions between the gene category and continuous independent variables. The results demonstrated statistically significant differences in gene expression divergence within the functional category of secretory genes related to genetic information processing, cellular processes, and environmental information processing (F4, 100 = 3.62, *p* < 0.001). This finding remained consistent even after accounting for the positive correlation between expression divergence and coding sequence divergence (GLM: F1, 100 = 5.4, *p* = 0.003; Figure 7C).

### 3.4. Correlation Between Expression Divergence, Coding Sequence Divergence, and Pleiotropy

**Figure 8:**
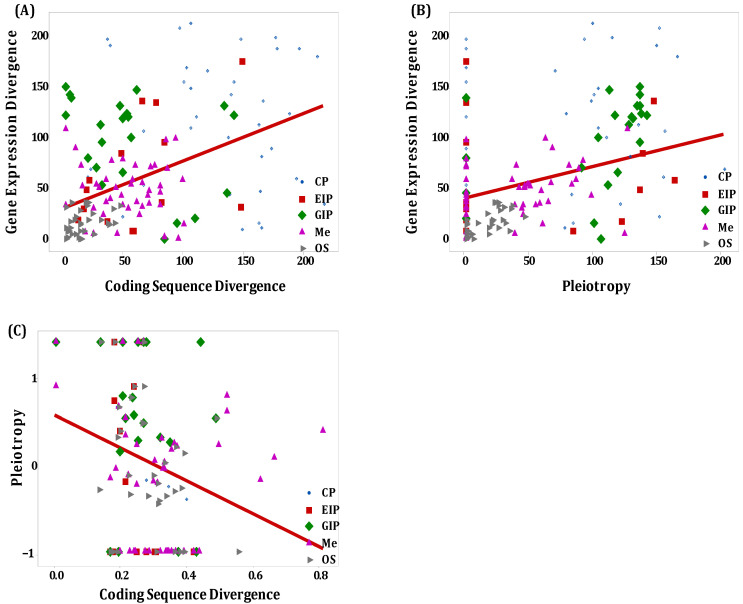
Schematic depiction of the relationship between expression divergence, coding sequence divergence, and pleiotropy among the functional categories of orthologous secretory genes; (**A**) Expression divergence against coding sequence divergence; (**B**) Expression divergence against pleiotropy; (**C**) Pleiotropy against coding sequence divergence.

The generalized linear models (GLMs) regarding both coding sequence and expression divergence revealed significant interactions among categories of secretory genes, expression divergence, sequence divergence, and pleiotropy, as previously noted. This finding suggests that the relationships between expression divergence and sequence divergence, expression divergence and pleiotropy, and coding sequence divergence and pleiotropy are influenced by the specific functional categories of secretory genes. To provide further insight into this issue, we calculated the Spearman rank correlation coefficients for expression divergence, sequence divergence, and pleiotropy across each distinct functional category of secretory genes (Figure 8).

The generalized linear models (GLMs) employed to investigate the divergence in coding sequences and expression demonstrated significant interactions among the categories of secretory genes, expression divergence, sequence divergence, and pleiotropy. This suggests a correlation between expression divergence and sequence divergence as well as between expression divergence and pleiotropy. Notably, there was a weak positive correlation between gene expression and sequence divergence in the categories of environmental information processing (rs = 0.418, *p* = 0.0172) and organismal systems (rs = 0.349, *p* = 0.00719). Conversely, a weak negative correlation was observed in genes involved in genetic information processing (rs = −0.422, *p* = 0.0447). However, no statistically significant interaction was found regarding expression and sequence divergence in secretory genes specifically associated with cellular processes (rs = −0.0727, *p* = 0.635) and metabolism (rs = −0.106, *p* = 0.340).

There was a moderate and positive correlation between expression divergence and pleiotropy for secretory genes related to organismal systems (rs = 0.631, *p* = 0.000112) and genetic information processing (rs = 0.582, *p* = 0.00355). However, no statistically significant correlation was found between expression divergence and pleiotropy for genes associated with cellular processes (rs = −0.144, *p* = 0.345), environmental information processing (rs = 0.0626, *p* = 0.734), and metabolism (rs = 0.172, *p* = 0.12). In contrast, a moderate and negative correlation was identified between coding sequence divergence and pleiotropy for secretory genes linked to cellular processes (rs = −0.523, *p* = 0.000226) and environmental information processing (rs = −0.515, *p* = 0.00256). Notably, there was no statistical correlation between coding sequence divergence and pleiotropy for genes related to genetic information processing (rs = −0.355, *p* = 0.0963), metabolism (rs = 0.0673, *p* = 0.546), and organismal systems (rs = 0.18, *p* = 0.176).

## 4. Discussion

The study revealed that genes associated with energy production and metabolism exhibited greater coding sequence divergence when compared with genes related to cellular processes, genetic information processing, environmental information processing, and organismal systems. In addition, secretory genes involved in metabolism also showed higher coding sequence divergence than their non-secretory counterparts. Notably, the variations in coding sequence divergence among different functional categories of secretory genes remained significant even after accounting for potential confounding factors, particularly gene expression divergence and pleiotropy. Furthermore, there are other factors that may contribute to the observed differences in the sequence divergence across the functional categories. An investigation into the divergence of genes reveals that those involved in secretion, in addition to those with restricted expression patterns, particularly within hepatic and mammary tissues, exhibit a greater degree of coding sequence divergence in comparison to non-secretory genes [29]. This divergence remains evident even when accounting for variables such as gene expression and pleiotropic effects. Given the substantial influence of the gene functional categories, it is likely that these categories also affect the divergence of the coding sequences. Specifically, the secretory genes implicated in metabolism demonstrated significantly higher rates of non-synonymous substitutions (dN), synonymous substitutions (dS), and the non-synonymous to synonymous substitution ratio (dNdS ratio) in comparison to genes belonging to other functional categories. 

The difference in coding sequence divergence among the functional categories of secretory genes was due to variations in selection pressure. Genes specifically encoding metabolism experienced either relaxed purifying selection or enhanced positive selection compared with genes implicated in environmental information processing, genetic information processing, and organismal systems. Research shows that selection pressures on coding sequences differ by gene category among species. In mammals, genes involved in immunity, reproduction, and secreted proteins evolve more rapidly and show signs of positive selection [30,31]. To differentiate between these explanations, it is necessary to employ tests using a codon model of sequence evolution. These tests require a larger dataset with more than two species. Codon models may be used to examine hypotheses regarding the selective pressures that influence sequence evolution and to pinpoint particular sites or lineages that have undergone adaptive evolution [32]. The study found evidence of positive selection of genes involved in metabolism. The secretory genes, which are directly engaged in metabolism, are most likely implicated in the response against stress, digestion, insect survival, and reproduction. Consistent with the current findings Dayı [33] reported that positive selection impacts metabolism and adaptation genes in insects and bats. Insects have genes related to digestion, oxidative reduction, transcription, and translation under positive selection. Reports on Alvinocaridid shrimps have shown that genes associated with energy metabolism frequently exhibit signs of positive selection. According to Wang et al. [34], these energy metabolism genes are significant in enabling shrimps to adapt to extremely harsh environments. Shapiro and Alm [35] also mentioned that genes involved in metabolism showed accelerated evolution in *Idiomarina loihiensis*. Thus, the substantial sequence divergence observed between *M. dirhodum* and *A. pisum* in metabolism-related genes is likely attributable to positive selection rather than relaxed constraints or negative selection. The rationale behind the positive selection of metabolic genes is not immediately clear. One possible explanation involves secretory genes that enable the recognition and exploitation of host resources, as well as mechanisms to avoid detection and suppress the immune responses of resistant plants. This could lead to rapid evolutionary changes in metabolic genes, resulting in a high evolutionary rate as these organisms adapt to counteract the adverse defense mechanisms of crops and environmental stressors. The coding sequences of metabolism-related genes showed a significant level of divergence, which likely contributed to the interspecific variation observed in the functional categories of secretory genes.

There are notable differences in the expression divergence among secretory genes that encode various functional categories. Specifically, genes associated with genetic information processing, metabolism, and cellular processes exhibited greater expression divergence compared with secretory and non-secretory genes related to environmental information processing. A study conducted by Gao et al. [36] analyzed differentially expressed genes and identified enriched pathways related to energy metabolism, developmental processes, and resistance to insecticides. However, the observed variations in mean expression between genetic information processing genes and other functional categories are likely influenced by the high variability in expression among different functional categories within the species in our dataset. After controlling for potential confounding factors, primarily coding sequence divergence, pleiotropy, and the interaction between gene category, sequence divergence, and pleiotropy, the differences in expression divergence among secretory genes linked to genetic information processing, cellular processes, and metabolism remained significant, showing only minor deviations in the strength of these differences across functional categories. Therefore, when accounting for confounding factors like coding sequence divergence and pleiotropy, along with the combined effects of sequence divergence and functional categories, the association between expression divergence and gene functional categories appears to be less pronounced.

The findings of the present study indicate that genes displaying accelerated diversification in coding sequences concomitantly manifest heightened diversification in expression patterns, augmented ratios of non-synonymous to synonymous substitution rates, diminished diversity at synonymous sites, and diminished pleiotropy in contrast to genes demonstrating less evolution. The analysis indicated a positive correlation between the divergence of coding sequences and the divergence of expression. Prior research examining the relationship between sequence divergence and expression divergence has produced conflicting results, with some studies concluding that no significant relationship exists between these two phenomena, thereby implying that the evolution of coding sequences and gene expression may constitute fundamentally independent processes [37]. In contrast, Hodgins KA [38] documented positive correlations between divergence in coding sequences and gene expression. Moreover, Warnefors and Kaessmann [39] observed that the correlation between sequence divergence and expression divergence varies across different organs, with the most robust correlation identified in the brain and the least pronounced correlation in the liver and testes. Nevertheless, the underlying reasons for this variability among the functional categories remain ambiguous. Various hypotheses regarding the correlation between sequence divergence and expression divergence include the potential influence of a shared factor affecting both realms, as demonstrated by investigations in both *Drosophila* and vertebrates [40,41]. Furthermore, the association may arise from analogous selection pressures that impact both coding sequences and the regulatory elements that govern gene expression. In certain cases, positive selection has been suggested as a crucial factor influencing the relationship between coding sequence divergence and expression divergence in insect species such as the housefly [41]. Conversely, relaxed purifying selection and genetic drift have been proposed as the primary determinants contributing to weaker positive correlations between gene coding sequence divergence and expression divergence [42]. The rationale behind these discrepancies among taxa remains indeterminate and may pertain to the efficacy of selection or the temporal extent since divergence, as well as variations in the rates of gene expression and sequence evolution over time, or disparities in the genomic targets of selection across distinct taxa.

The interaction of genes can significantly impact how selection affects the variability of their sequences. Notably, genes exhibiting pleiotropic effects, which influence the expression of multiple other genes, may undergo stronger selective pressures [43]. Our research used co-expression networks to examine the extent of pleiotropy among secretory genes and its relation to the coding sequence divergence in the rose grain aphid. We discovered a negative correlation between pleiotropy and coding sequence divergence. This finding can be attributed to the more intense negative selection and coding sequence divergence acting on highly connected genes, even after accounting for variations in gene expression divergence. Conversely, local regulatory variation in gene expression divergence appeared to correspond with reduced positive selection and enhanced gene connectivity (pleiotropy). According to Barbitoff, et al. [44], genes characterized by high pleiotropy are typically expressed extensively across functional groups, participate in numerous biological processes, and exhibit a greater frequency of protein–protein interactions. These genes are subjected to more rigorous negative selection pressures; however, they also display indicators of recent positive selection, implying their involvement in adaptive mechanisms.

Variations in coding sequences and the expression of orthologs genes can result in differences in the functions of secretory genes, which are influenced by gene duplication and the development of specialized roles in paralogs. Li et al. [45] established a connection between the age of duplicate genes and their divergence in expression, although the specifics of this relationship are not fully understood. Gene duplication typically increases the expression divergence, facilitating specialization in various tissues or developmental stages. Insects often experience elevated rates of gene duplication, leading to significant functional variations that are not directly linked to evolutionary timelines. Some gene families show rapid structural changes driven by natural selection [46]. proposed that duplication rates differ across functional categories of secretory genes, but thorough analysis requires a high-quality reference genome, which is currently unavailable for this study. Exploring the relationship between gene duplication and the divergence of coding sequence and expression could yield valuable insights into the evolution of secretory gene functions.

## 5. Conclusions

This research sheds light on the coding sequence divergence of genes linked to metabolism compared with other functional categories. It highlights how gene functional categories influence evolutionary trajectories, particularly pointing out that metabolic secretory genes experience a more significant coding sequence divergence due to varying selection pressures. The presence of positive selection on these genes indicates their adaptation to environmental challenges and interactions with hosts, potentially driving rapid evolutionary shifts. The research also explores the link between coding sequence divergence and expression divergence, uncovering a positive correlation that supports some existing studies while contradicting others. This complexity underscores the intricate nature of gene evolution, where both coding sequences and expression patterns are influenced by a combination of selection pressures, gene connectivity, and functional roles. Furthermore, the study of pleiotropy suggests that genes with high connectivity may face stronger negative selection, adding another dimension to the evolutionary landscape. The possibility of gene duplication facilitating functional specialization among paralogs also warrants further investigation, especially given the high incidence of gene duplication in insects. Future research should incorporate larger datasets and more sophisticated codon models to better comprehend the selective pressures across different taxa and functional categories. Overall, this research enriches the discourse on molecular evolution by detailing the dynamic interplay between coding sequence divergence, expression divergence, and the impact of functional categories. These findings enhance our understanding of evolutionary mechanisms and pave the way for future studies into the complexities of gene evolution in response to environmental and biological pressures.

## Figures and Tables

**Table 1:** Number of putative secretory genes associated with each functional category.

Replication	KEGG Pathways	Classification of SG Based on KEGG	SG Annotated in RG Aphid and Pea Aphid	Relative Number of Genes Used for Sequence Divergence
Cellular Processes	333	52	35	35 (10.51%)
Environmental Information Processing	319	17	8	8 (2.51%)
Genetic Information Processing	808	21	19	19 (2.35%)
Metabolism	778	56	28	28 (3.59%)
Organismal Systems	384	18	10	10 (2.60%)
Total	2622	164	100	100 (3.81%)

**N.B.**: SG classification based on KEGG refers to the categorization of secretory genes according to KEGG, while RG Aphid denotes the rose grain aphid.

## Data Availability

Supplementary files of research articles titled “Divergence in coding sequences and expression patterns among the functional categories of secretory genes between two aphid species”. The raw sequencing data and information generated from this study are deposited in the NCBI SRA (http://www.ncbi.nlm.nih.gov/bioproject/PRJNA1134911) and zendo (https://zenodo.org/uploads/13530906) website portals.

## Supplementary Materials

The following supporting information can be downloaded at: https://www.mdpi.com/article/10.3390/biology14080964/s1, FASTA 1: CDS of non-secretory gene sequences of rose grain and pea aphids; FASTA 2: CDS of secretory gene sequences of rose grain and pea aphids; Table: Supplementary files for coding sequence and expression divergence.
